# FBXW7 circular RNA regulates proliferation, migration and invasion of colorectal carcinoma through NEK2, mTOR, and PTEN signaling pathways in vitro and in vivo

**DOI:** 10.1186/s12885-019-6028-z

**Published:** 2019-09-13

**Authors:** Haoran Lu, Baofu Yao, Xinyuan Wen, Baoqing Jia

**Affiliations:** 1 Department of General Surgery, Chinese PLA General Hospital, No. 28 Fuxing Road, Haidian District, Beijing, 100853 China; 2 Department of General Surgery, Affiliated Hospital of Jining Medical University, Jining, Shandong Province China; 3 Department of Oncological Surgery, Beijing Geriatric Hospital, Beijing, China

**Keywords:** Colorectal cancer, circRNAs, Circ-FBXW7, Cell proliferation, Migration, Invasion

## Abstract

**Backgrounds:**

A number of circular RNAs (circRNAs) have been identified in various cancer including F-box and WD repeat domain containing 7 (FBXW7) circular RNA (circ-FBXW7), which can suppress glioma cell growth. However, the role of circ-FBXW7 in colorectal cancer (CRC) remains unclear. We aimed to investigate the effect and mechanisms of circ-FBXW7 on CRC progression.

**Methods:**

The expression of circ-FBXW7 in CRC patients was detected by PCR. Stably knockdown of circ-FBXW7 (si circ-FBXW7) cell lines and overexpression of circ-FBXW7 (oe circ-FBXW7) cell lines were constructed by small interfering RNA method and plasmids transfection in CRC SW480 and SW620 cells. The functional experiments including cell proliferation, migration and invasion were carried out by cell counting kit-8 (CCK-8) assay, wound healing assay and trans well assay. The xenograft animal models were established to evaluate the effect and the underlying molecular mechanisms of circ-FBXW7 on CRC progression.

**Results:**

CRC samples had a significantly lower level of circ-FBXW7 compared to normal tissue. si circ-FBXW7 notably promoted the proliferation, colony formation, cell migration and invasion of CRC cell in vitro. On contrast, circ-FBXW7 overexpressed significantly suppressed CRC cell proliferation, migration and invasion. Similarly, si circ-FBXW7 stimulated the tumor growth and circ-FBXW7 overexpression repressed the tumor progression in SW480 and SW620 tumor models, which suggested that circ-FBXW7 could serve as a target biomarker of CRC. Further study found that si circ-FBXW7 up-regulated the mRNA and protein expressions of NEK2 and mTOR, and diminished the PTEN expression. Whereas, overexpressed circ-FBXW7 induced the tumor suppression via reversing the expressions of NEK2, mTOR, and PTEN.

**Conclusion:**

circ-FBXW7 plays a major role in controlling the progression of CRC through NEK2, mTOR, and PTEN signaling pathways and may be a potential therapeutic target for CRC treatment.

**Graphical abstract:**

Circ-FBXW7 controls the progression of CRC through NEK2, mTOR, and PTEN signaling pathways and its overexpression inhibits colorectal cancer cell migration and invasion, suggesting the potential therapeutic target for CRC treatment. 
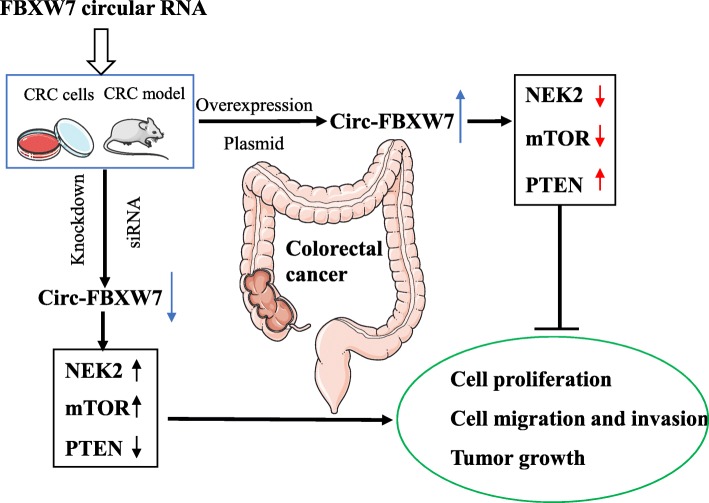

## Background

Colorectal cancer (CRC) is the fourth diagnosed cancer and the second leading cause of cancer-related death with increasing morbidity in the population [1]. Despite recent improvements in early diagnosis and the development of more effective treatments for CRC, some of CRC patients are failed to respond to the treatments. Furthermore, the newest targeted therapeutic drugs do not cure the patient or induced the drug resistance [2]. Thus, it is critical for improving understanding of the underlying cellular basis of CRC to the development of novel therapeutics drugs. In the past decades, numerous factors including gene mutation, aberrant DNA methylation, microRNA (miRNA) [3], noncoding RNA deregulation, and circular RNAs (circRNAs) [4] are involved in CRC [5]. As a large proportion of the non-cording RNA family, circRNAs are closely associated with a variety of human diseases including Alzheimer’s disease, cardiovascular disease, as well as cancers. circRNAs are found in many kinds of tumors such as CRC, gastric cancer, and hepatocellular carcinoma, and can be used as the potential biomarker for cancer diagnosis or as predictive marker for patient outcome [4]. Ten circRNAs are involved in the regulation of cell proliferation, migration, and invasion in CRC [6].

F-box and WD repeat domain containing 7 (FBXW7), also known as FBW7 and CDC4, is a p53-dependent tumor suppressor frequently mutated in human cancers, including 15–20% of CRC. FBXW7 plays the role in controlling cell metastasis, stress responses, and immune functions at so on [7], which relates to regulate the crucial onco-proteins such as cyclin E, Notch, and c-Myc [8]. Loss of FBXW7 greatly accelerates tumorigenesis, which results in a more cancer cell proliferative and less cell differentiation in CRC, hepatocellular carcinoma, gastric carcinoma, breast cancer, and so on [9]. Recent clinical study showed that FBXW7 missense mutations portend a worse prognosis in patients with metastatic CRC [10]. Mutations of FBXW7 also mediated intrinsic and acquired resistance of CRC to targeted agents by blocking Mcl-1 degradation [11]. However, FBXW7 could significantly inhibit the CRC cell migration and invasion by targeting the HIF1α/ CEACAM5 axis. Patients with high expression of FBXW7 in CRC gave better 5-year overall survival (OS) and disease-free survival (DFS) than that of low expression [12].

Recently, it was reported that circular RNA form of the FBXW7 (circ-FBXW7) and its encoded protein FBXW7–185 aa could suppress glioma cell proliferation by arresting cell cycle. Circ-FBXW7 might have potential prognostic implications in glioma, which had an increased total survival time in higher circ-FBXW7 patients [13]. However, to date, roles and mechanisms of circ-FBXW7 in CRC remain to be elucidated. Our current study determined the expression of cicr-FBXW7 in CRC patients and established circ-FBXW7 overexpression as well as knockdown SW480 and SW620 cell lines to investigate the effect of circ-FBXW7 on CRC progress. We also verified the function of circ-FBXW7 on regulation of tumor growth and the potential target proteins in SW480 and SW620 tumor models. This study would provide new insights into circ-FBXW7 interaction with CRC progression.

## Methods

### Clinical specimens

All CRC tissues and normal mucosal tissue obtained from the colon cancer patients or rectal cancer patients (*n* = 20) were collected from General Hospital of the People’s Liberation Army. The collected tissues were stored in a − 80 °C refrigerator for PCR analysis [13].

### Cancer cell culture and transfection

Human CRC SW480 (ATCC® CCL-228™) and SW620 (ATCC® CCL-227™) cell lines were purchased from American Type Culture Collection (ATCC, USA) in April 2018. SW480 cells were cultured in DMEM with 10% fetal bovine serum (FBS) and 1% penicillin-streptomycin, SW620 cells were grown in RPMI1640 with 10% FBS and 1% penicillin-streptomycin. Cells were maintained at 37 °C in a humidified incubator contacting 5% CO_2_.

For the knockdown study, specific mall interfering RNAs (siRNA) against circ-FBXW7 were designed and constructed from Biomics Biotechnologies (Jing su, China) using the following sequences: 5′-CCAUGCAAAGUCUCAGAAU-3′ and transfected into colorectal cancer cells (SW480 and SW620) by Lipofectamine 2000 Transfection Reagent (Invitrogen, USA) according to the manufacturer’s instructions. For the overexpression of circ-FBXW7, SW480 and SW620 cells were transduced with circ-FBXW7 overexpressing plasmid, which was bought from Beijing polepolar Biotechnology (Beijing, China). To prove the success of the construction, cells were collected and then detected the circ-FBXW7 mRNA and protein levels by qRT-PCR and western blot after 48 h transfection. The transfected cell lines were tested as being mycoplasma free by using PCR Mycoplasma Test Kit II (AppliChem) and authenticated by examination of morphology and consistent in vitro performance.

### Cancer cell growth assays

In our study, cell viability was analyzed by cell counting kit-8 (CCK-8) assay. Both of SW480 and SW620 cancer cells were divided into three groups including the wild type (WT), siRNA circ-FBXW7 (si circ-FBXW7) and circ-FBXW7 overexpressing (oe circ-FBXW7). 2 × 10^3^ cancer cells were cultured in 96-well plate for 24 h (*n* = 6) and 10 μL of CCK-8 solution were added to the cancer cells. Then the cancer cells were incubated for another 1 h at 37 °C in dark. The optical density (OD) of cell lysates was measured at 450 nm by a microplate reader. The experiments were performed in triplicated and repeated for three times.

The cell proliferation was detected by colony formation assay. The same cancer cell groups (WT, si circ-FBXW7, and oe circ-FBXW7, 500 cells) were planted into 6-well plate and cultured in medium containing 10% FBS for 14 days. After washing with PBS supplemented with methanol, the cells were stained with 0.1% crystal violet solution. The clone number was counted manually.

### Cell invasion assays

Cell invasion of SW480 and SW620 cells was detected by Trans well chambers (pore size: 8 μm, BD Biosciences) with Matrigel according to the manufacturer’s instructions. 1 × 10^5^ cells with serum-free culture medium (DMEM or RPMI-1640) were seeded into the upper chamber, and culture medium containing 10% FBS was added to the lower chamber. Invaded cells were fixed, stained and the numbers of invasion cells were detected using the microscope.

### Cancer cell migration assay

Same groups including WT, si circ-FBXW7, and oe circ-FBXW7 in SW480 and SW620 cells (2 × 10^5^) were seeded in six-well plates. After 100% confluence, the cells were scraped with a pipette tip for cell migration assay. Culture medium with 10% FBS was changed every day to remove detached and damaged cells. The width of cell migration was monitored by microscopy at 0, 24, 48, 72, and 96 h. Cell migration was calculated an average width of the cell wound. The cell migration rate was calculated by the following formula: Rate (%) = (W_0_-W_t_/W_0_) × 100, W_0_ was the cell width at 0 h; W_t_ was the cell width at different time (24–96 h). The experiment was conducted in triplicate.

### Tumor xenograft assay

Animal xenograft to investigate the antitumor effect of circ-FBXW7 in vivo. Thirty-six female BALB/c nude mice (SPF, 4–6 weeks, 18–22 g) were bought from Beijing Vital River Laboratory Animal Technology Co., Ltd., and they were permitted to acclimate to the environment for 7 days before the experiments began. All the mice were kept under temperature-controlled conditions, underwent a reverse dark-light cycle and were provided standard mouse pellets and tap-water ad libitum in individually ventilated cages (IVC) with autoclaved bedding in the laboratory. The mice were randomly divided into six groups (*n* = 6) including WT group, si circ-FBXW7 group and oe circ-FBXW7 group for injection SW480 cancer cells and SW620 cancer cells, respectively. A density of 1 × 10^7^/mL siRNA circ-FBXW7 (si circ-FBXW7) cell lines, circ-FBXW7 overexpressing (oe circ-FBXW7) cell lines or normal cells (WT) of SW480 and SW620 were separated injected subcutaneously into the right flank of mice with 0.2 mL. The tumor growth in each mouse was monitored every 2 day by measuring the width (W) and length (L) of tumor with Vernier caliper. The tumor volume (V) was calculated by the following formula: V (mm^3^) = (W × L^2^)/2. Three weeks after cancer cells injection, all the mice were euthanized by a cervical vertebrae luxation, and the tumors were removed and weighted. The study protocol was approved by the Institutional Animal Care and Use Committee of Chinese Academy of Medical Sciences Institute of Radiation Medicine.

### Western blot analysis

The total proteins were isolated from cancer cells or tumor tissues with RIPA lysis buffer. The protein concentrations were detected by BCA Protein Assay Kit. All the proteins were separated through sodium dodecyl sulphate-polyacrylamide gel electrophoresis (SDS-PAGE), and then transferred to polyvinylidene fluoride (PVDF) membranes. After blocking with 5% nonfat milk for 2 h, the membranes were incubated with indicated primary antibodies overnight at 4 °C. The membranes were washed with tris-buffered saline with tween-20 (TBST) and incubated with secondary antibody for 1 h. At last, the protein bands were detected with the chemiluminescence system (Bio-Rad Laboratories Inc., CA). β-actin was used as an internal reference.

### Quantitative real-time polymerase chain reaction (qRT-PCR)

Total RNA from cancer cells or tumor tissues was extracted with Trizol reagent (Invitrogen). Complementary DNA (cDNA) was used as reverse transcriptase according to the protocol provided by Takara reverse transcription kit (Takara, China). NanoDrop 2000 (Quawell, USA) was available to analyze the concentrations and quality of RNA. The cDNA was amplified by SYBR Green qRT-PCR Master Mixed (Thermo-Fisher Scientific) with ABI 7500 thermal cycler (Applied Biosystems, CA). We used β-actin as an internal control. Primer sequences were showed as following: NEK2 (5′-CCTGGAGCAGAAGGAACGTG-3′ and 5′-TGGCTGAGGATGGAAGATCAAG-3′), mTOR (5′-AGAGGTCGGCACTCCACTAT-3′ and 5′-TGGCCAGGCTTCTGAACAAA-3′), PTEN (5′-AGCCTCTTGATGTGTGCATT-3′ and 5′-CCATTGGTAGCCAAACGGAAC-3′) β-actin (5′-GCCCTGAGGCTCTCTTCCA-3′ and 5′-GCGGATGTCGACGTCACA-3′). The gene expression level was determined based on the 2^-∆∆Ct^ method.

### Statistical analysis

We analyzed the experiment data with SPSS 20.0 software (SPSS, Chicago, IL, USA) and GraphPad Prism 7 (GraphPad Software, La Jolla, CA, USA). Results are shown as the mean ± standard deviation (SD). Student *t* test and one-way ANOVA were utilized to analyze significant difference. A *p*-value < 0.05 was considered as statistical significance.

## Results

### The expression of circ-FBXW7 in clinical CRC patients

**Fig. 1:**
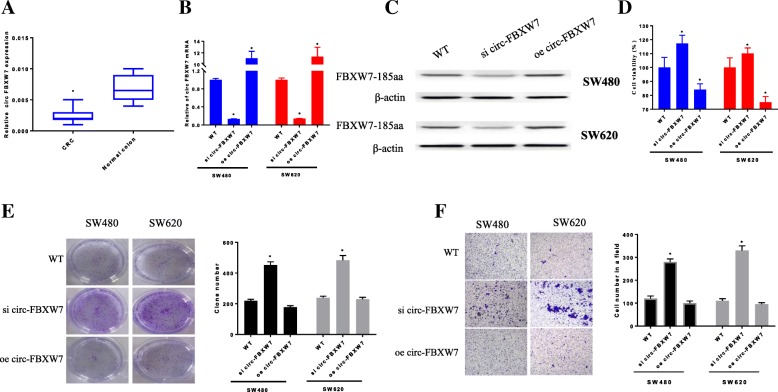
Expression levels of circ-FBXW7 in CRC samples and normal colon tissues (*n* = 10) were detected by PCR (**a**). Circ FBXW7 mRNA (**b**) and proteins (**c**) expressed in SW480 and SW620 cancer cells and effect of circ-FBXW7 on colon cancer cells behavior. **d** Cell viability analysis using CCK-8 assay. **e** Effect of siRNA or overexpression of circ-FBXW7 on SW480 and SW620 cancer cell colony formation capacity. The graph is the summarized data of the colony formation assay. **f** Cell invasion ability analysis was detected by trans wells method. *p < 0.05 vs. Normal colon group or WT group

The expression of circ-FBXW7 was detected in ten pairs of cancerous and adjacent noncancerous tissues derived from CRC patients. In CRC tissues, circ-FBXW7 expression was lower compared with that in paired adjacent noncancerous tissues (*P* < 0.05, Fig. 1a). This result suggested that circ-FBXW7 is indeed related to the CRC progression.

### Circ-FBXW7 inhibited the CRC cell proliferation

The knockdown and overexpression of circ-FBXW7 were established in SW480 and SW620 cancer cell lines. As shown in Fig. 1b and c, the level of circ-FBXW7 mRNA was sharply decreased after si RNA transfection, and it was remarkably improved after the overexpression by plasmid transfection in SW480 and SW620 cancer cell. Similarly, the protein level of FBXW7–185 aa was downregulation in si circ-FBXW7 cells and upregulation in oe circ-FBXW7 cells. Cells in the wild type (WT) group did not receive any treatment and served as a negative control.

When compared with the WT group, SW480 cell viability was suppressed in oe circ-FBXW7 group, whereas it was significantly improved in si circ-FBXW7 group (*p* < 0.05). Similar results were achieved in SW620 cells (Fig. 1d). The results of clone information also revealed that both of SW480 and SW620 cells were markedly increased in si circ-FBXW7 group (*p* < 0.05), while cell proliferation was comparable between the oe circ-FBXW7 and WT groups (Fig. 1e and f). The promotion of CRC cell proliferation after circ-FBXW7 silencing indicated that circ-FBXW7 played a role in regulating cell proliferation in CRC.

### Circ-FBXW7 suppressed CRC cell migration and invasion

To determine whether circ-FBXW7 affected the mobility of CRC cells, we assessed the migratory and invasive abilities of SW480 and SW620 cells at 48 h after transduction with oe circ-FBXW7 or si circ-FBXW7. Trans well assays demonstrated that si circ-FBXW7 remarkably enhanced the invasive capabilities of SW480 and SW620 cells compared with the WT group. Also, there are no difference between oe circ-FBXW7 and WT group (Fig. 1f).

**Fig. 2:**
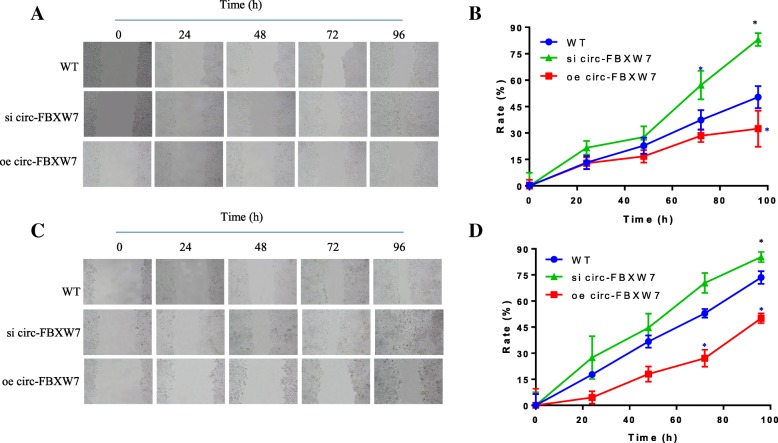
Effects of circ-FBXW7 overexpression and knockdown on SW480 and SW620 cells migration assay. Wounds were made with a pipette tip in confluent monolayers. **a** representative SW480 cancer cell wound healing width images of 0, 24, 48, 72, and 96 h. **b** represented the SW480 cancer cell migration rate, which was calculated according to the width from 0 h to 96 h. **c** representative SW620 cancer cell wound healing width images of 0, 24, 48, 72, and 96 h. **d** represented the SW620 cancer cell migration rate, which was calculated according to the width from 0 h to 96 h. Data are mean ± SD. *p < 0.05 vs. WT group

Further, we detected the effect of circ-FBXW7 on cell migration by scratch wound healing assay. The width of healed wound in WT group was gradually decreased from 0 h to 96 h. Increased cell motility was observed in si circ-FBXW7 CRC cell lines, while oe circ-FBXW7 suppressed the migration rate (Fig. 2). These results suggested that circ-FBXW7 repressed the CRC cell migration and invasion.

### Circ-FBXW7 attenuated CRC cell progress in vivo

**Fig. 3:**
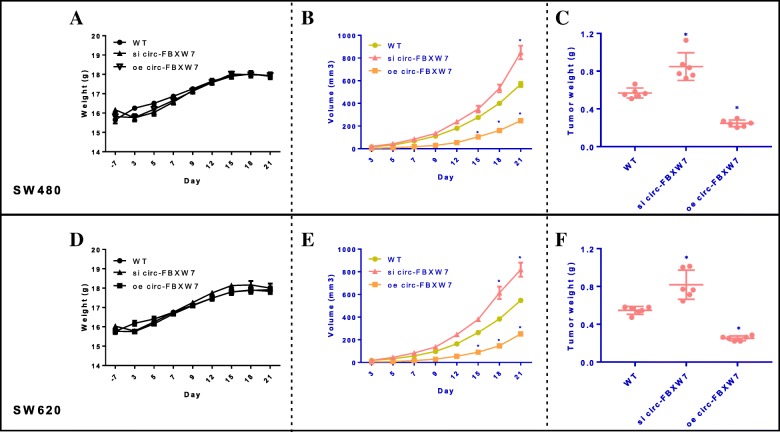
Overexpression of circ-FBXW7 inhibits colorectal cancer growth, and knockdown of circ-FBXW7 promotes colorectal cancer growth in vivo. All of **a**, **b**, and **c** represented the indexes for SW480 tumor models; **d**, **e**, and **f** were the indexes of SW620 tumor models. **a** and **d** the body weights of mice were weighted during the experiment. **b** and **e** The volumes of Xenografts were measured every 2 days for 21 days. **c** and **f** Tumor weight was taken from nude mice after 21 days of growth. * *p* < 0.05 vs. WT group

The in vitro experiments revealed that knockdown of circ-FBXW7 promoted CRC cells growth, migration and invasion, but circ-FBXW7 overexpression reversed the cancer cell behaviors. Then we wonder whether circ-FBXW7 inhibited CRC cell progression in vivo. Nude mice were injected with si circ-FBXW7 CRC cell line, oe circ-FBXW7 CRC cell line, WT CRC cells, and then tumor growth was monitored during 3 weeks. Mice body weights were not influenced in the three groups in SW480 and SW620 tumor models (Fig. 3a and d). circ-FBXW7 overexpression in SW480 significantly decreased tumor volume during the experiment (Fig. 3b), which leading to a smaller tumor weight than model group (0.25 g vs 0.57 g) (Fig. 3c), and the inhibition rate of oe circ-FBXW7 group was reached to 56.51%. si circ-FBXW7 in SW480 animal model exhibited a reversed tendency on tumor growth with 0.85 ± 0.13 g of tumor weight (growth rate was 49.34%). Consistently, oe circ-FBXW7 also dramatically attenuated the tumor growth in SW620 tumor model with 0.25 ± 0.02 g of tumor weight (vs 0.55 ± 0.04 g, WT group), while si circ-FBXW7 markedly promoted the tumor growth with the tumor weight of 0.82 ± 0.14 g (Fig. 3e and f). These results showed that circ-FBXW7 inhibited CRC propagation in vivo.

### Circ-FBXW7 regulated the expressions of NEK2, mTOR, and PTEN in CRC models

**Fig. 4:**
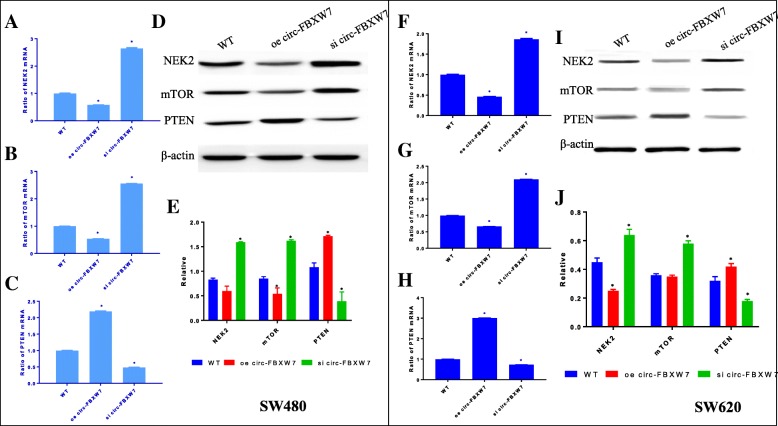
Levels of NEK2, mTOR, and PTEN mRNAs and proteins were determined by qRT-PCR and western blot in tumor tissues. Effect of circ-FBXW7 on mRNA expressions of NEK2 (**a**), mTOR (**b**) and PTEN (**c**) as well as the them proteins expression (**d**) and quantitative analysis (**e**) in SW480 tumor. Overexpressed circ-FBXW7 could repressed the NEK2 and mTOR expressions but improved PTEN expression. Circ-FBXW7 showed similar effect on mRNA expressions of NEK2 (**f**), mTOR (**g**) and PTEN (**h**) as well as the proteins (**i** and **j**) in SW620 tumor tissues. Data are expressed as mean ± SD. * *p* < 0.05 vs. WT group

To explore the potential underlying molecular mechanism of circ-FBXW7 in regulating CRC cell growth, the expression levels of NEK2, mTOR, and PTEN were examined by qRT-PCR and western blot methods in SW480 and SW620 tumor tissues (Fig. 4). Results showed that si circ-FBXW7 up-regulated the mRNA and protein levels of NEK2 and mTOR, but diminished PTEN expressions in SW480 and SW620 tumors. On the contrary, oe circ-FBXW7 down-regulated the mRNA and protein levels of NEK2 and mTOR, and enhanced PTEN expression in the two tumor models, which further confirmed that si circ-FBXW7 promoted CRC progression through upregulation of NEK2 and mTOR, and downregulation of PTEN.

## Discussion

The application of the aberrant epigenetic modifications as diagnostic, prognostic and predictive biomarkers for CRC have been reported in numerous studies, which open new avenues for exploration of reliable and robust biomarkers to improve the management of CRC patients. Epigenetic biomarkers for CRC including methylated DNA, miRNAs, and blood-based methylation markers were found in clinical or experiment tests [14]. Many types of circRNAs have been found in a variety of tumors, and may relate to cancer cell proliferation, metastasis, invasion or as potential biomarkers for detecting cancer [4, 15]. It was reported that 11 circRNAs were upregulated and 28 circRNAs were downregulated in CRC tissues [6], of which the most significantly downregulation circRNA was derived from PTK2 tumor suppressor gene [16]. Recently, several circRNAs were found to be involved in the proliferation and metastasis of CRC, such as circular RNA circPPP1R12A [17], circCCDC66 [18], circRNA_104916 [19], and circ_0055625 [20]. These findings provided valuable insights into the development of novel potential therapeutic targets or biomarkers for CRC. Yang et al. reported that circ-FBXW7 as well as the encodes protein FBXW7–185aa have potential prognostic implications in brain cancer [13]. FBXW7 as a potent tumor suppressor is one of the most common mutated genes in human cancers, which inhibits the progression of tumors by targeting specific substrates for ubiquitination and proteasomal degradation [21, 22]. Loss or mutation of FBXW7 has been found in multiple human tumors including CRC, which suggest it can be as an independent prognostic marker in some tumors [23, 24]. Our study first found that circ-FBXW7 was low expressed in CRC patients, and then we evaluated the effect of circ-FBXW7 on CRC by establishing the overexpressed and knockdown of circ-FBXW7 in SW480 and SW620 cancer cell lines. We used siRNA method to knockdown of circ-FBXW7 to loss of function and transfection plasmid to overexpress of circ-FBXW7 to gain of function. Our results showed that knockdown of circ-FBXW7 promoted SW480 and SW620 cells proliferation, migration, invasion, and the tumor growth. On the contrary, circ-FBXW7 overexpression inhibited CRC cells proliferation and migration as well as the growth of tumor weight.

As reported, circ-FBXW7 is abundantly expressed in the normal human brain, while it is reduced in clinical malignant glioma patients, and it is positively associated with glioblastoma patient OS [13]. Consistently, low expression of circ-FBXW7 was found in CRC patients according to our study. Circ-FBXW7 exhibited the same phenomenon to CRC cells by in vitro and in vivo experiments. si circ-FBXW7 expression in SW480 and SW620 cells significantly improved the cell proliferation, colony formation, cell migration and invasion, whereas, the overexpression of circ-FBXW7 reversed the changes, which are consistent with FBXW7 functions in regulating the cancer cellular processes [8, 11, 12, 23, 25]. In addition, some researchers found that the FBXW7 is a binding target protein like an intermediate adjustment involving several signaling pathways. mTOR is a major target for treatment human diseases including cancer. It is reported that loss of FBXW7 and deletion or mutation of PTEN can activate mTOR [26]. Our study showed that the levels of mTOR were significantly enhanced in si circ-FBXW7 groups of SW480 and SW620 cells, while PTEN were significantly decreased. On the contrary, circ-FBXW7 overexpressed reduced mTOR expression and improved the expression of PTEN. Similar to FBXW7, PTEN is also a tumor suppressor and predictive marker for CRC patient outcome, which can induce the cell resistance [27]. Furthermore, our findings showed that the level of NEK2 was remarkably elevated in si circ-FBXW7 CRC cell groups, but decreased in oe circ-FBXW7 CRC cell groups. NEK2 regulates tumor progression, drug resistance and tumorigenesis, which is considered to be a potential biomarker of cancers [28]. The upregulation of NEK2 is linked to poor prognosis for CRC patients [29], while NEK2 siRNA may be a useful method for treatment colorectal patients [30]. These results indicate that circ-FBXW7 is a necessary factor in controlling the CRC cell process by regulating cancer cell generation and metastasis through inhibiting the NEK2, mTOR signal pathways and activating PTEN.

## Conclusion

Our results revealed that circ-FBXW7 plays a tumor suppressor role in CRC. Silence expression of circ-FBXW promoted the CRC cell proliferation, migration and invasion, as well as the tumor growth. Forced expression of circ-FBXW7 inhibited CRC progressing, which is associated with NEK2, PTEN, and mTOR signaling pathways. Therefore, circ-FBXW7 can be a potential circRNA target for treatment of CRC.

## Data Availability

The datasets used and/or analyzed during the current study are available from the corresponding author on reasonable request.

## Abbreviations

circ-FBXW7: FBXW7 circular RNA
circRNAs: Circular RNAs
CRC: Colorectal cancer
FBXW7: F-box and WD repeat domain containing 7
mTOR: Mammalian target of rapamycin
NEK2: NIMA-related kinase 2
PTEN: Phosphatase and tensin homolog
